# Crystal structure of Bax bound to the BH3 peptide of Bim identifies important contacts for interaction

**DOI:** 10.1038/cddis.2015.141

**Published:** 2015-07-09

**Authors:** A Y Robin, K Krishna Kumar, D Westphal, A Z Wardak, G V Thompson, G Dewson, P M Colman, P E Czabotar

**Affiliations:** 1 The Walter and Eliza Hall Institute of Medical Research, Melbourne, VIC, Australia; 2 Department of Medical Biology, The University of Melbourne, Melbourne, VIC, Australia

## Abstract

The BH3-only protein Bim is a potent direct activator of the proapoptotic effector protein Bax, but the structural basis for its activity has remained poorly defined. Here we describe the crystal structure of the BimBH3 peptide bound to BaxΔC26 and structure-based mutagenesis studies. Similar to BidBH3, the BimBH3 peptide binds into the cognate surface groove of Bax using the conserved hydrophobic BH3 residues h1–h4. However, the structure and mutagenesis data show that Bim is less reliant compared with Bid on its ‘h0' residues for activating Bax and that a single amino-acid difference between Bim and Bid encodes a fivefold difference in Bax-binding potency. Similar to the structures of BidBH3 and BaxBH3 bound to BaxΔC21, the structure of the BimBH3 complex with BaxΔC displays a cavity surrounded by Bax *α*1, *α*2, *α*5 and *α*8. Our results are consistent with a model in which binding of an activator BH3 domain to the Bax groove initiates separation of its core (*α*2–*α*5) and latch (*α*6–*α*8) domains, enabling its subsequent dimerisation and the permeabilisation of the mitochondrial outer membrane.

The intrinsic pathway to apoptosis is regulated by interactions between members of three factions of the Bcl-2 protein family: the BH3-only proteins such as Bim and Bid, which initiate the process, the essential effectors Bax and Bak, and the prosurvival members, which oppose the action of both other factions.^1^ The interactions between prosurvival Bcl-2 family members and BH3 peptides have been well characterised as the earliest studies with Bcl-x_L_ and a BakBH3 peptide.^2^ Such complexes are readily formed in solution by incubating the C-terminally (ΔC) truncated prosurvival Bcl-2 protein with a BH3 peptide. The absence of the C-terminal segment that can anchor the Bcl-2 protein in a membrane apparently has little effect on the ensuing complex. That complex is believed to be responsible for the antiapoptotic function of Bcl-2, by sequestration of the BH3 motif either of the so-called BH3-only proteins such as Bim ('mode 1') or of Bax or Bak ('mode 2').^3^

Although proapoptotic Bax and Bak have very similar three-dimensional structures to their prosurvival relatives,^4, 5, 6^ until recently^7, 8^ no structure of a complex of either Bax or Bak with a BH3 peptide had been captured, despite an accumulation of evidence that Bax and Bak could be activated directly by interaction with the BH3-only proteins Bid, Bim and possibly others.^9, 10, 11, 12, 13^

Unlike Bak, which is constitutively anchored in the mitochondrial outer membrane (MOM) via its C-terminal segment, Bax is largely cytosolic in healthy cells and accumulates at the MOM only upon a death signal.^14, 15^ There it is believed to display at least two different conformers,^16, 17^ one loosely associated with the MOM and another in which its membrane anchor (helix *α*9) is inserted into the MOM. In striking contrast to the antiapoptotic relatives of Bcl-2, a construct of Bax lacking its C-terminal membrane anchor, BaxΔC21, has no measurable interaction with BH3 peptides. However, in the presence of the detergent octylglucoside binding is detected by surface plasmon resonance (SPR) for the BH3 peptides of Bim, Bid, Bak and Bax itself with IC50s in the range of 0.1–1μM,^7, 18^ some 100-fold weaker compared with those measured similarly with (for example) Bcl-x_L_ΔC, where no detergent is required. Weaker interactions between BidBH3 or BimBH3 and BaxΔC as compared with Bcl-x_L_ΔC are not inconsistent with various models for the function of the Bcl-2 protein family whereby the prosurvival molecules sequester BH3 motifs with high affinity and long half-lives, but proapoptotic Bax and Bak are activated by transient (‘hit-and-run') interactions with BH3 motifs.^19, 20, 21^

Complexes of BaxΔC21 bound to BH3 peptides from Bid and Bax have been prepared by coincubation of the protein with CHAPS and an excess of the peptides.^7^ Under these conditions, the protein undergoes a conformational change and dimerises via domain swapping of helical segments *α*2–*α*5 and *α*6–*α*8, dubbed ‘core' and ‘latch' domains, respectively. Although this ‘core/latch dimer' is thought to be an *in vitro* artefact, its formation is diagnostic for the core and latch separation, which is required for membrane-associated Bax to dimerise via its core domains and then to permeabilise the MOM.^7^ If the latch domain is absent, as in a recombinant construct of GFP fused to Bax *α*2–*α*5, the core domain forms BH3:groove symmetric dimers,^7^ which, consistent with a wide body of evidence,^21, 22, 23, 24, 25^ are present in apoptotic pores.

Previous work^7^ highlighted the importance of two hydrophobic ‘h0' residues (Figure 1) in the peptide (I82/I83 in BidBH3) in governing Bid's ability to activate Bax. Similar to Bid, Bim is also a potent direct activator of Bax, and the ‘h0' amino acids in Bim are proline and glutamic acid. In the absence of a structure of BimBH3:BaxΔC, it remained unclear how these ‘h0' residues were accommodated. Here we describe the crystal structures of BimBH3 26- and 20-mer peptides bound to BaxΔC26. Comparison with the structure of BidBH3:BaxΔC21 allows a dissection of the critical contacts between these two peptides and BaxΔC. The binding profiles of mutant BH3 peptides illustrate that BimBH3 binding to Bax is less dependent on the ‘h0' residues compare with that in the case for BidBH3. The BimBH3 complex displays a similar cavity adjacent to Bax *α*1, *α*2, *α*5 and *α*8 as seen in the BidBH3 complex. We also describe a structure of BidBH3 bound to a BaxΔC21 mutant, I66A, which is more typical of the BH3 signature of antiapoptotic Bcl-2 family proteins^7, 26^

## Results

### BimBH3:BaxΔC26 crystal structure

**Figure 1:**
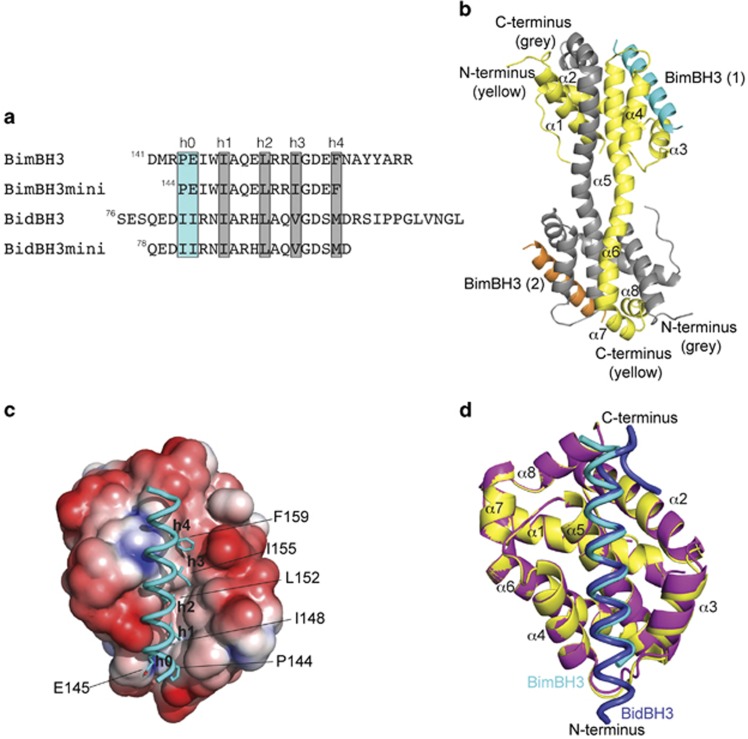
BimBH3 binds BaxΔC. (**a**) BH3 peptide sequences used in this study, indicating the 5 hydrophobic amino-acid positions ‘h0'–‘h4'. (**b**) The core/latch dimer of BaxΔC26 bound to BimBH3. The two Bax polypeptides, shown here as cartoons, are coloured yellow and grey, and the two Bim peptides cyan and orange. A crystallographic dyad symmetry axis passes through the centre of this particle. (**c**) Structure of BimBH3:BaxΔC26 complex. The globular unit depicted comprises Bax residues 1–128 from one polypeptide and 129–166 from the other, together with the associated Bim peptide. Bax is represented by its surface and colour coded according to surface charge (blue, positive potential (4*kT*/*e*); red, negative potential (−2*kT*/*e*); calculated using the Adaptive Poisson–Boltzmann Solver.^41^ The trace of the Bim peptide (cyan) is shown with ‘h0' (P144, E145), ‘h1' (I148), ‘h2' (L152), ‘h3' (I155) and ‘h4' (F159) represented as sticks. (**d**) Overlay of BimBH3:BaxΔC26 with BidBH3:BaxΔC21 (PDB:4BD2). Structures represented as cartoon ribbons, yellow for Bax in the Bim complex and magenta for Bax in the Bid complex. The peptides (Bim cyan and Bid blue) stand vertically in the foreground in this view (similar to Figure 1c), with their N termini at the bottom of the figure

Activation of BaxΔC21 by BimBH3 or BidBH3 peptides (Figure 1a) in the presence of CHAPS yields a core/latch dimer of BaxΔC21,^7^ which can be purified on size exclusion chromatography (SEC). BidBH3:BaxΔC21 complex crystals were then obtained by complementing the dimer with an excess of BidBH3 peptide before crystallisation. The resulting structure contained the core/latch dimer in complex with two BidBH3 peptides.^7^ Although this strategy failed to give crystals of BimBH3:BaxΔC21, we noted that the C terminus of the BaxΔC21 construct was disordered in previously published structures (PDB codes 4BD2 and 4BD6). We therefore made a complex instead with a BaxΔC26 construct, this crystallised in different conditions to those used for the BidBH3:BaxΔC21 complex. The solved structure revealed one-half of a core/latch dimer and one BimBH3 peptide within the asymmetric unit (Figure 1b), similar to those of BaxΔC21 complexes with BaxBH3 and BidBH3.^7^

Aligning the globular entity comprising Bax residues 10–128, the Bim peptide and Bax residues 129–166 from the partner polypeptide in the core/latch dimer with the comparable structure of the BidBH3:BaxΔC21 (PDB code 4BD2) illustrates that the BimBH3 peptide engages the canonical peptide-binding groove of BaxΔC (Figure 1c) and that the structures of BaxΔC in the two complexes are near identical (Figure 1d). However, F30 within the Bax protein core displays different conformations in the two complexes. In the BimBH3 complex, its phenyl ring has elevated *B*-factors. Unmodelled difference electron density adjacent to F30 in the BimBH3 complex suggests that this anomaly is due to the different crystallisation conditions used for the two complexes (see Materials and Methods) rather than a consequence of differences between the Bim and BidBH3 sequences. A small shift in the position of Bax helix *α*3 between the two structures (Figure 1d) may be because of the contacts between Bim W147 and Bax M79. The C terminus of BaxΔC26 (G166) is visible together with one residue from the vector (serine), whereas the C-terminal residues 168–171 of BaxΔC21 in the BidBH3 complex are disordered.^7^ The Bim and Bid peptides differ in length (Figure 1a), but the structures of the helical segments from residues ‘h0'–‘h4' are in close alignment. The C-terminal residues of the Bim peptide (–R–R– Figure 1a) are disordered.

### Role of BimBH3 ‘h0' residues in Bax activation

**Figure 2:**
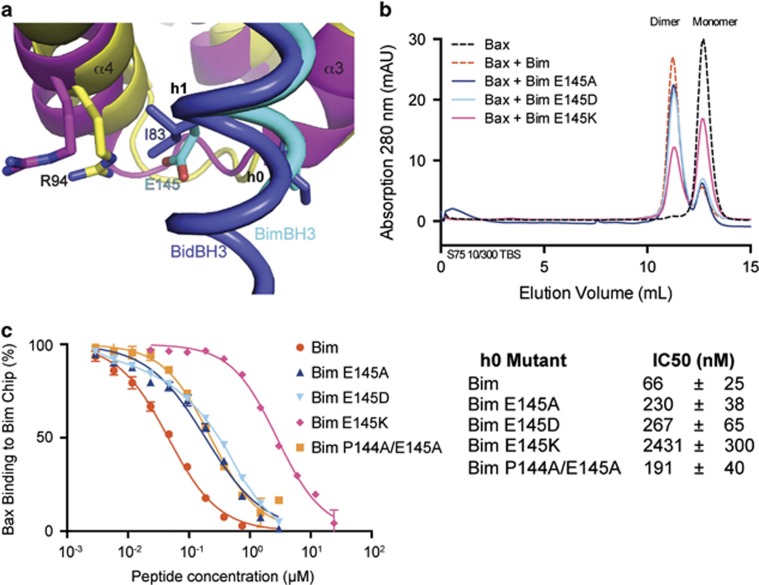
Accommodation of E145 in the ‘h0' position of BimBH3. (**a**) Enlargement of Figure 1d in the region of Bax residue R94. (**b**) Gel filtration profiles of BaxΔC treated (or not) with CHAPS and BimBH3 variants. Only the charge-swapped BimBH3 mutant E145K appears seriously compromised in its capacity to unlatch Bax and promote formation of the core/latch dimer. (**c**) SPR measurements of BimBH3 and BimBH3 mutant peptides displacing BaxΔC from a BimBH3 chip. The derived IC50 values are tabulated

Alanine substitutions at the ‘h0' position in BidBH3 (I82/I83) (Figure 1a) abolish its ability to bind BaxΔC21 in SPR assays, to trigger formation of core/latch dimers of BaxΔC21 and to permeabilise liposomes exposed to full-length Bax.^7^ The first of the ‘h0' residues of BimBH3, P144, makes similar hydrophobic contacts with Bax as its counterpart I82 in BidBH3. The alkyl moiety of BimBH3 residue E145 occupies the same space as the BidBH3 residue I83, but its carboxylate moiety does not form a typical pair of hydrogen bonds with the guanidinium of Bax R94 (Figure 2a). The importance of the ‘h0' residues of BimBH3 in the interaction with Bax was explored by mutagenesis.^7^ We tested the ability of ‘h0' BimBH3 mutants to trigger core/latch dimer formation of BaxΔC21 or bind to BaxΔC21. BimBH3 mutants E145A and E145D were less able to dimerise BaxΔC21 (Figure 2b) and showed reduced binding to BaxΔC21 in the SPR assays (Figure 2c). The activity of the double mutant BimBH3 P144A/E145A was not reduced significantly further. The BimBH3 charge swap mutant E145K mutant was the most crippled of those tested, reducing BaxΔC21 dimerisation by >50% (Figure 2b) and binding some 40-fold more weakly to BaxΔC21 (IC50 of 2.4*μ*M) (Figure 2c). Thus, unlike the loss-of-function BidBH3 mutation I82A/I83A, in BimBH3 the P144A/E145A mutant is only marginally compromised.

### Differences in the binding of BimBH3 and BidBH3 to BaxΔC21

**Figure 3:**
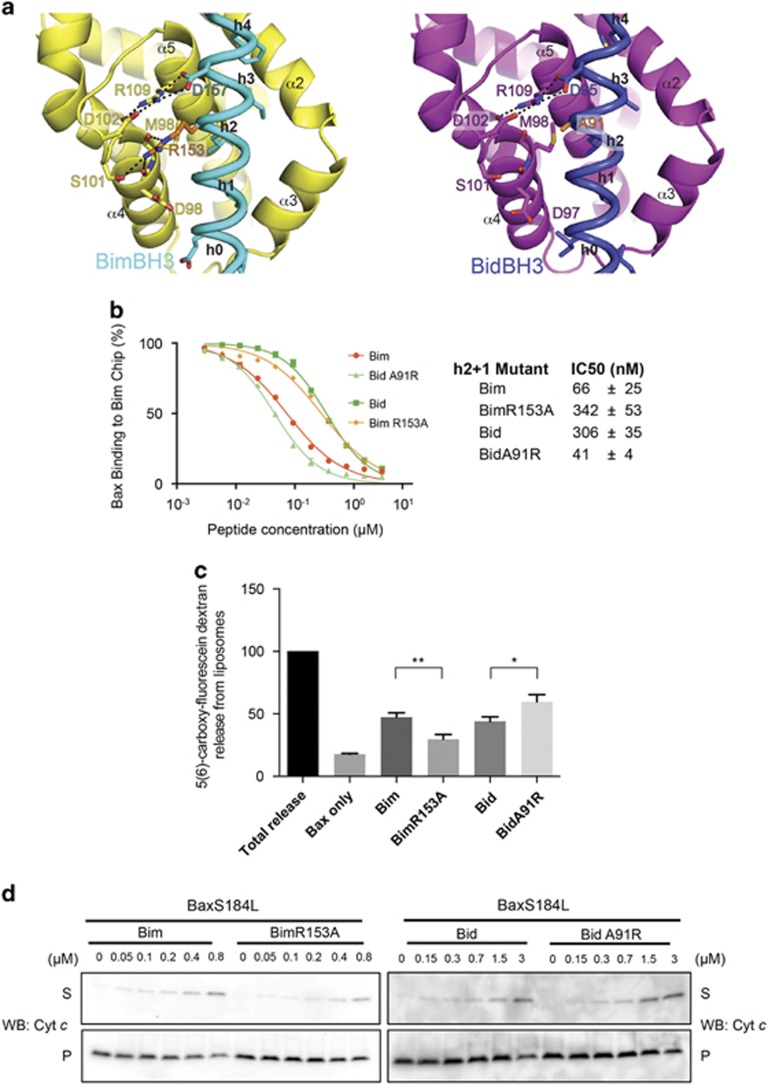
Comparison of BimBH3 and BidBH3 complexes with BaxΔC. (**a**) BimBH3:BaxΔC26 (left) and BidBH3:BaxΔC21 (PDB:4BD2, right) showing interactions between Bim R153 (orange sidechain) and Bax that are absent in the Bid complex where the peptide residue ‘h2+1' is A91. (**b**) SPR measurements of BimBH3, BidBH3 and mutant peptides displacing BaxΔC from a BimBH3 chip. The derived IC50 values are tabulated. (**c**) Liposome permeabilisation by full-length Bax in response to BimBH3, BidBH3 and mutant peptides (error bars represent S.D. of three independent experiments. * *P*<0.05 and ** *P*<0.005. (**d**) Cytochrome *c* release assays do not reflect the fivefold differences in binding from panel b. BidBH3 A91R was at best a marginally more effective than BidBH3 at releasing cytochrome *c* from mitochondria isolated from MEFs expressing BaxS184L. Similarly, BimBH3 R153A shows very little reduction in potency over BimBH3 at releasing cytochrome *c* from MEFs displaying Bax. Data are representative of two independent experiments

We further compared the BimBH3:BaxΔC26 and the BidBH3:BaxΔC21 (PDB code 4DB2) structures to identify peptide–protein interactions specific to either complex. One significant structural difference is at position ‘h2+1', R153 in Bim and A91 in Bid (Figure 1a). In the BimBH3 complex structure (Figure 3a, left panel), Bim R153 forms a planar stack,^27^ with the interactions network formed by Bim D157, Bax R109 and Bax D102. Bim R153 is also hydrogen bonded with Bax S101 and the backbone carbonyl groups of Bax D97 and M98. In the BidBH3 complex structure (Figure 3a, right panel), Bid A91 cannot form similar interactions.

To determine if the ‘h2+1' position influences the binding of BimBH3 or BidBH3 to BaxΔC21, we tested the activity of mutant BH3 peptides BimBH3 R153A and BidBH3 A91R. Their ability to dimerise BaxΔC21 was largely unaffected (data not shown). In the SPR assay (Figure 3b), BimBH3 bound BaxΔC21 some fivefold more tightly than BidBH3, and the BimBH3 R153A mutant behaved like BidBH3, whereas the BidBH3 A91R mutant behaved similar to BimBH3. In liposome permeabilisation assays (Figure 3c), BimBH3 had a greater capacity to activate Bax compared with BimBH3 R153A, whereas BidBH3 A91R had enhanced activity over BidBH3. To test whether this change in binding affinity influenced apoptotic activity, we assessed the ability of each peptide to induce cytochrome *c* release from mitochondria isolated from *Bak*^−/−^ *Bax*^−/−^ mouse embryonic fibroblasts (MEFs) ectopically expressing the mitochondrially targeted Bax variant BaxS184L.^28^ The trends observed in the liposome assays were not apparent in these cytochrome *c* release experiments (Figure 3d), possibly due to limitations in experimental sensitivity. The presence of endogenous prosurvival proteins in the experiment may have also impacted on the result.

### Comparison of the complexes of BimBH3 with Bax and Bcl-x_L_

**Figure 4:**
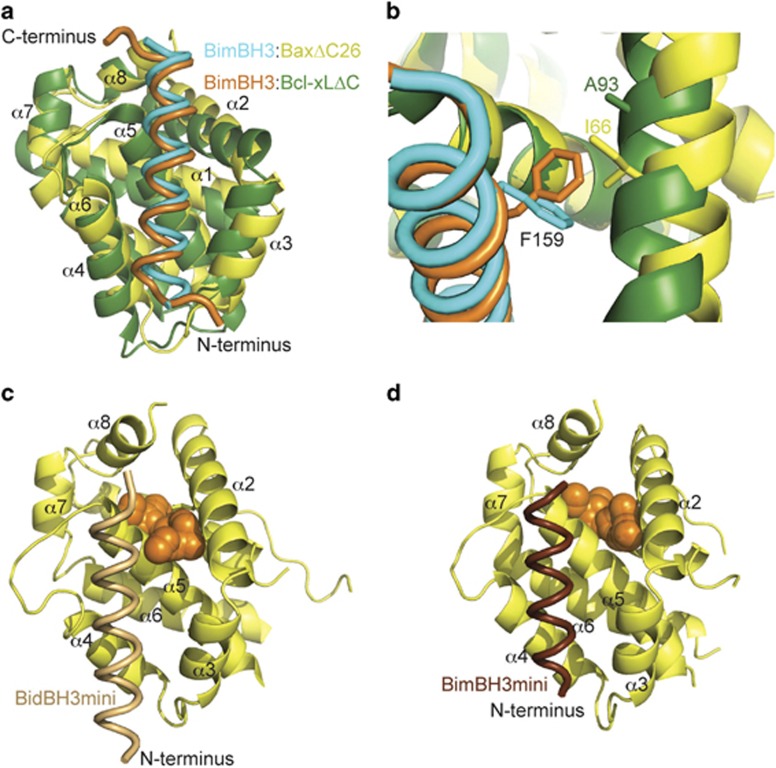
Comparison of BimBH3 binding to prosurvival and proapoptotic Bcl-2 family proteins. (**a**) Alignment of BimBH3:Bcl-xLΔC (orange/dark green) with BimBH3:BaxΔC26 (cyan/yellow). (**b**) Detail from panel (**a**) of Bim F159 in the two complexes, showing also A93 from Bcl-xL and I66 from Bax. Colour code as in panel (**a**). (**c**) Cavity (orange) in the BidBH3mini:Bax complex. (**d**) Cavity in BimBH3mini:Bax complex

The structure of BimBH3:Bcl-x_L_ΔC^29^ was aligned with the structure of the Bax moiety of BimBH3:BaxΔC26 (Figure 4a). The BimBH3 peptide is helical until residue Y163, as previously observed in complexes of BimBH3 with prosurvival molecules.^29, 30, 31^ As reported earlier from a similar comparison of the BidBH3:BaxΔC21 and BimBH3:Bcl-x_L_ΔC^7^, the Bim peptide in Bax is displaced ~1.6 Å towards its C terminus with respect to its position in the prosurvival protein complex. Another feature of this alignment consistent with our earlier analysis is the observation that Bax *α*2 and *α*3, on the right hand side of the peptide in Figure 4a, are more distant from the peptide compared with their prosurvival protein counterparts. In particular, in the BaxΔC complex Bim ‘h2' (L152) and ‘h3' (I155) residues are contacted only by Bax amino acids L70 and L76 from *α*2 and *α*3, whereas in the Bcl-x_L_ΔC complex at least five amino acids (F97, Y101, A104, F105 and L108) from *α*2 and *α*3 of Bcl-x_L_ participate in interactions with these two Bim residues. Note also that BimBH3 ‘h4' residue F159 adopts different rotamers in the two complexes (Figure 4b), in the case of Bax making a stacking interaction with I66 and in the case of Bcl-x_L_ making an edge contact with A93.

### BH3mini complexes with BaxΔC26 and BaxΔC28

We previously showed that mini-BH3 motifs, 20-mers spanning residues ‘h0'–‘h4', sufficed to trigger dissociation of BaxΔC into core and latch domains.^7^ To confirm that the N- and C-terminal segments of the longer peptides did not influence the peptides' interaction with Bax, we crystallised both BimBH3mini and BidBH3mini with BaxΔC26. These structures are essentially identical to that reported above and to BidBH3:BaxΔC21 (PDB 4BD2^7^), except to note that in the case of the BimBH3mini complex, no clear density is evident for F159 (the ‘h4' residue). Whereas in the BidBH3:BaxΔC21 structure the C terminus of the peptide is passively folded back (PDB code 4BD2), the use of a shorter peptide leaves the top of the groove (between Bax helices *α*2, *α*5 and *α*8) open. Note also that the conformation of F30 in the BimBH3mini:BaxΔC26 complex resembles that found in all of the BidBH3 complexes with Bax that we have studied, further suggesting that the altered orientation of this residue in the BimBH3:BaxΔC26 structure stems from the different crystallisation conditions.

The BH3mini:BaxΔC26 complexes contain a cavity between helices *α*1, *α*2, *α*5 and *α*8 (shaped with the side chains of residues 26, 30, 60, 63, 66, 67, 110, 111, 114, 115, 158 and 161) as observed also in the BidBH3:BaxΔC21 complex but not in the apo BaxΔC21 core/latch dimer.^7^ The cavity volumes, as calculated on the CASTp server,^32^ are, respectively, 123Å^3^ and 111 Å^3^ in the BidBH3mini:BaxΔC26 (Figure 4c) and the BimBH3mini:BaxΔC26 (Figure 4d), similar in size to the BidBH3:BaxΔC21 cavity of 140Å.^3^

**Figure 5:**
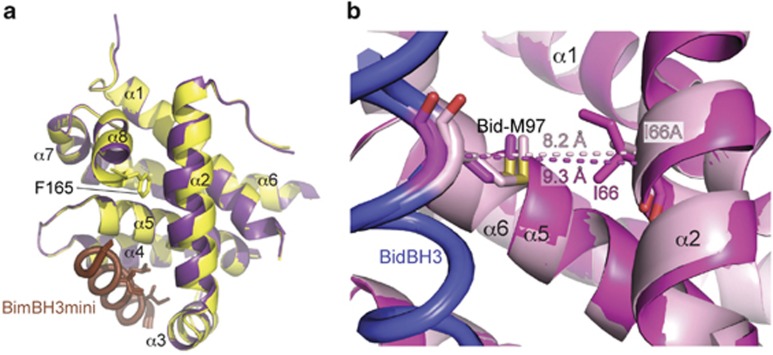
Interplay between helices 2 and 8 in peptide:Bax complexes. (**a**) Overlay of BimBH3mini:BaxΔC26 (yellow) and BimBH3mini:BaxΔC28 (purple) showing collapse of helix 2 against the truncated helix 8 of Bax. (**b**) Overlay of BaxI66A (salmon) and Bax wild-type (pink) bound to BidBH3. Distances between the C*α* atoms of Bax residues I/A66 and Bid residue M97 are indicated

To explore the consequences of removing *α*8, presumably an early event in separation of the core and latch domains, we crystallised the BimBH3mini:BaxΔC28 complex and determined its structure. C-terminal truncations that remove more of *α*8 cannot be expressed.^7^ Although no significant differences were observed in the way the peptide interacts with the protein, the absence of F165 from Bax in the structure of BimBH3mini:BaxΔC28 allows Bax *α*2 to move towards *α*8 by up to 1Å (Figure 5a.) This movement of Bax *α*2 partly fills the cavity observed in the BaxΔC26 complexes described above.

### BidBH3:BaxI66AΔC21

Earlier work had demonstrated that BidBH3 M97 contacts Bax I66, and alanine mutants at either or both of these positions reduced the capacity of the peptide to release the core domain from the latch domain.^7^ To investigate whether this contact was responsible for the apparent outwards displacement of Bax *α*2 in its complex with BH3 peptides relative to the position of the Bcl-x_L_ *α*2 in equivalent complexes, we crystallised a core/latch dimer complex of BaxI66AΔC21 with BidBH3. That structure is very similar to the wild-type complex, the largest difference being that the C terminus of the Bid peptide is disordered in the crystal, rather than folding back over Bax I66A as it does with Bax I66. In addition, a slight ‘collapse' of Bax *α*2 towards the core of the complex (Figure 5b) is evident in a 1 Å shortening of the distance between the C*α* atoms of Bid M97 and Bax I66A (8.2 Å) as compared with Bax I66 (9.3 Å). As a consequence, the cavity in the BidBH3:BaxI66AΔC21 structure is reduced to a size not significantly larger than cavities found (for example) in the interior of the complex between BimBH3 and Bcl-x_L_.

## Discussion

In previous work, we described the interactions between BaxΔC21 and the BidBH3 and BaxBH3 peptides.^7^ Crystal structures of these complexes contained core/latch dimers of BaxΔC21 with one BH3 peptide bound to each of the canonical BH3-binding grooves in the Bax dimer. The structures led to the realisation of the importance of hydrophobic amino acids at the so-called ‘h0' position in the BH3 sequence, the observation of internal cavities within the structures and other distinctive characteristics of the complexes of BH3 peptides with Bax as compared with prosurvival Bcl-2 family members. However, missing from that study was a structure of the important activator BH3-only protein Bim in complex with BaxΔC.

### The ‘h0' motif and the relative potencies of Bim and Bid for BaxΔC

The ‘h0' residues of Bim are less critical for activating Bax compared with their counterparts in Bid. The double mutant P144A/E145A has an ~3-fold loss of affinity for BaxΔC, whereas the Bid double mutant I82A/I83A failed to bind to BaxΔC in our assay.^7^ The structures reported here also define the role of the ‘h2+1' residue in the BH3 motif in determining the difference in affinity of Bim and BidBH3 peptides for BaxΔC21. That residue in Bim (R153) makes several additional interactions with BaxΔC21 in the *α*4–*α*5 loop region and accounts for an ~5-fold tighter binding of BaxΔC21 to BimBH3 than to BidBH3. Differences in potency of this order have been reported for Bim- and Bid-induced activation of Bax,^33^ and we have observed similar differences in Bax-binding assays reported here. However, this difference did not equate to a significant difference in the ability of Bid or Bim to preferentially induce Bax-mediated mitochondrial permeabilisation (Figure 3d). Although our data are inconclusive on the question of the biological relevance of these differences, our results support a model in which activation is mediated by a BH3 motif engaging with the canonical BH3-binding groove of Bax. Our studies do not directly address activation of Bax by BimBH3 at a site remote from the groove proposed by others.^34, 35^

### Internal cavities

Experimental evidence indicates that internal cavities in proteins are destabilising.^36^ The BimBH3mini:BaxΔC26 structure contains a cavity of similar location and shape to that observed in the published structures with Bid and Bax peptides (PDB codes 4BD2, 4BD6). Although smaller in volume compared with the analogous cavity in the BidBH3:BaxΔC21 structure, the residues surrounding the cavities are similar. In the BimBH3 complex, they are F30, L63, I66, G67, V110, V111, F114, Y115, W158 and L161. Of these, only I66 directly contacts the bound BimBH3 peptide, but peptide-binding does require a large movement of helix 3 and the C-terminal end of helix 2 from their position in the apo BaxΔC21 structure.^7^ F30 is among those residues whose backbone amide resonance is grossly affected by titration of the BaxBH3 peptide into BaxΔC21.^37^ We interpret the presence of cavities within the core of Bax when bound to an activator BH3 peptide as a sign of instability of the complex, pre-empting the release of the Bax latch domain (*α*6–*α*8) as required for the core domains (*α*2–*α*5) to dimerise.^7^ Note that we have calculated the cavity using the structure of the BimBH3mini:BaxΔC26 complex, because the anomalous conformation of F30 in the BimBH3:BaxΔC26 described above obscures the effect. The loss of the cavity in BimBH3mini:BaxΔC28 complex through movement of *α*2 demonstrates that the positions of *α*2 and *α*8 are coupled, but sheds little additional light on the unfolding pathway of unlatching. It has been suggested, based on molecular modelling, that when Bim binds to Bax, it displaces helix 8 owing to a clash between C-terminal regions of the BimBH3 and T167 on Bax.^31^ No such displacement was observed here, although the peptide we have used may not be quite long enough for a rigorous test of that proposal. Removal of helix 8 at some stage during Bax activation is clearly a requirement of our hypothesis that the core domain must be unlatched to allow oligomerisation.^7^

### Comparison with BimBH3 bound to Bcl-x_L_ΔC

Structural overlays of BidBH3:BaxΔC21 and BaxBH3:BaxΔC21 with prosurvival proteins bound to BH3 peptides suggested that the peptide was slightly displaced towards its C terminus in the BaxΔC21 complex compared with the prosurvival protein complexes.^7^ Accompanying this displacement was the observation that Bax helices *α*2 and *α*3 were more distant from the bound BH3 peptides compared with its Bcl-x_L_ counterpart. A similar feature is now evident in the comparison of BimBH3 peptides bound to Bcl-x_L_ΔC and BaxΔC. These anomalies, which may derive in part from the deletion of two residues connecting helices 2 and 3 in Bax as compared with Bcl-x_L_,^7^ offer a partial explanation for the weaker binding of Bim to Bax as compared with prosurvival proteins.

## Conclusions

Bim and Bid are both potent direct activators of Bax, but Bim is less dependent on interactions via its ‘h0' residues than is Bid. The fivefold greater potency of BimBH3 compared with BidBH3 for binding BaxΔC was shown to be attributable to a single residue in BimBH3, although this difference did not translate into a significant difference in capacity to release cytochrome *c* from mitochondria in our assays. Activation of Bak by BH3 peptides shows marked similarities to that of Bax.^38^ Core/latch dimers of Bax and Bak form on treatment of the monomeric proteins with certain BH3 peptides and CHAPS, and Bax and Bak core domains form symmetric homodimers, supporting that unlatching the core domain is a necessary step to Bax and Bak oligomerisation and permeabilisation of the MOM.

## Materials and Methods

### Reagents, peptides and recombinants proteins

Lipids were purchased from Avanti Polar Lipids, Inc. (Alabaster, AL, USA). Detergents were purchased from Affymetrix (Santa Clara, CA, USA). Peptides were purchased from Mimotopes (Notting Hill, VIC, Australia) (BimBH3, BimBH3mini, BimBH3 E145A, BimBH3 P144A/E145A, BimBH3 R153A, BidBH3, BidBH3mini, BidBH3 A91R) or from GenScript (Piscataway Township, NJ, USA) (BimBH3 E145D, BimBH3 E145K). Three different constructs of Bax, each containing the mutations C62S and C126S and individually truncated at the C terminus by 21, 26 and 28 amino acids, were expressed and purified according to a described protocol.^6^ These are, respectively, referred to as BaxΔC21, BaxΔC26 and BaxΔC28. All three constructs were cloned into pTYB1; owing to a cloning artefact, BaxΔC26 and BaxΔC28 contain two extra residues (Ser–Ser) at their C termini after intein digestion. Proteins were expressed in *Escherichia coli* BL21(DE3) or ER2566 cells, lysed in TBS (20 mM Tris (pH8), 150 mM NaCl) and purified into two steps: chitin affinity chromatography followed by SEC (Superdex75) in TBS.

### Core/latch dimer preparation

Core/latch dimers induced by the several BH3 peptides mutants were prepared according to a similar protocol to that previously described.^7^ BaxΔC21 (2 mg/ml) was mixed with a twofold molar excess of BH3 peptides in TBS in the presence of 1% CHAPS. The mixture was incubated for 1 h at room temperature and analysed by SEC (Superdex75 column in TBS) to evaluate the potency of the BH3 peptide to dimerise BaxΔC21.

### Bax binding

Bax binding assays were performed using a protocol modified from that described previously.^18^ BaxΔC21 at 25 nM was incubated with increasing concentrations of BH3 peptides in SPR buffer (10 mM HEPES, 150 mM NaCl, 3.4 mM EDTA, 0.005% Tween-20, pH 7.4) containing 0.5% octylglucoside. Samples were injected onto a CM5 chip bearing immobilised BimBH3 and inert Bim4E peptides. Specific BimBH3 binding responses were assessed by subtracting Bim4E channel responses from the BimBH3 channel responses. IC50 values were calculated with GraphPad Prism (GraphPad Software, La Jolla, CA, USA).

### Liposomes

Liposome release assays were performed using a modified version of that described previously.^11^ Liposomes consisting of 46% phosphatidylcholine, 25% phosphatidylethanolamine, 11% phosphatidylinositol, 10% phosphatidylserine and 8% cardiolipin encapsulating self-quenching 5(6)-carboxy-fluorescein were prepared by drying lipid mixes in chloroform and 0.01% butylated hydroxytoluene under N_2_ and then resuspending in SUV buffer (10 mM HEPES (pH 7.5), 135 mM KCl, 1 mM MgCl_2_) containing 50 mM 5(6)-carboxy-fluorescein. The suspension was extruded through a 100 nm pore size membrane and then passed over a PD10 column to remove unincorporated dye. Liposomes at 4 *μ*g/ml were incubated with 50 nM full-length Bax and 500 nM BH3 peptide in SUV buffer for 1 h at RT. Fluorescence of released self-quenching 5(6)-carboxy-fluorescein was measured with an excitation wavelength of 485 nm and emission wavelength of 535 nm. Full-length Bax for these experiments was expressed and purified as described above.

### Cytochrome *c* release

BaxS184L was stably expressed in *Bax*^−/−^ *Bak*^−/−^ MEFs and cytochrome *c* release assays were performed as described for Bak.^23^ Supernatant and pellet fractions were separated and analysed for cytochrome *c* by immunoblotting.

### Crystallisation, data collection and processing

The core/latch dimers of BaxΔC21, BaxΔC26 or BaxΔC28 were induced by BimBH3 or BidBH3 peptide as described before and purified by SEC.^7^ The purified dimer was concentrated (5 mg/ml) and supplemented with threefold molar excess peptide 30 min before crystallisation tray setup. BidBH3, BimBH3mini or BidBH3mini peptides in complex with the core/latch dimer crystals of BaxI66AΔC21, BaxΔC26 or BaxΔC28 grew in conditions similar to 1 M tri-sodium citrate and 0.1 M sodium cacodylate (pH 6.5) at 277 K. Crystals were frozen into liquid nitrogen using well solution complemented with 20% ethylene glycol. Crystallisation conditions for the BimBH3:BaxΔC26 were 0.1 M sodium bicine (pH 9.0), 20% PEG6000 at 277 K and crystals were frozen in well solution complemented with 20% ethylene glycol. Data were collected on the MX2 beamline at the Australian Synchrotron (Clayton, VIC, Australia) at 100 K and processed using XDS.

### Crystallography

All structures were solved by molecular replacement in Phenix^39^ using the core/latch dimer of BidBH3:BaxΔC21 as a model (PDB code 4BD2). The final models for all structures were achieved through several cycles of building in Coot^40^ and refinement in Phenix. Structure pictures were created using PyMOL (The PyMOL Molecular Graphics System, Version 1.5.0.4; Schrödinger, LLC). Structural alignments were performed in PyMOL. Cavities were detected with the CASTp server^32^ using the default probe size (1.4 Å). Coordinates and structure factors have been deposited in the Protein Data Bank with accession codes: 4ZIE (BimBH3:BaxΔC26), 4ZIF (BimBH3mini:BaxΔC26), 4ZIG (BidBH3mini:BaxΔC26), 4ZIH (BimBH3mini:BaxΔC28) and 4ZII (BidBH3:BaxI66AΔC21). Table 1 shows the crystallographic data collection and refinement statistics.

## Figures and Tables

**Table 1:** Data collection and refinement statistics

	BimBH3:BaxΔC26	BimBH3mini:BaxΔC26	BidBH3mini:BaxΔC26	BimBH3mini:BaxΔC28	BidBH3:BaxI66AΔC21
Wavelength (Å)	0.9537	0.9537	0.9537	0.99537	0.953687
Resolution range (Å)	33.97–1.797 (1.861–1.797)	19.77–2.401 (2.486–2.401)	19.96–2.2 (2.278–2.2)	19.68–2.5 (2.589–2.5)	46.42–2.191 (2.269–2.191)
Space group	P 43 21 2	P 43 21 2	P 43 21 2	P 43 21 2	P 43 21 2
Unit cell dimensions (Å)	95.44 95.44 36.35	96.14 96.14 37.26	100.12 100.12 37.41	95.73 95.73 37.1	103.801 103.801 38.01
Total reflections	228 519 (22 368)	55 437 (5686)	78 642 (7899)	48 467 (4765)	151 151 (12 901)
Unique reflections	16194 (1580)	7219 (706)	10108 (986)	6334 (609)	11053 (1031)
Multiplicity	14.1 (14.2)	7.7 (8.1)	7.8 (8.0)	7.7 (7.8)	13.7 (12.5)
Completeness (%)	99.92 (99.43)	99.92 (100.00)	99.96 (100.00)	99.98 (99.84)	98.87 (94.33)
Mean *I*/sigma(I)	30.73 (2.57)	22.48 (2.61)	15.18 (2.61)	18.46 (2.09)	17.29 (1.63)
Wilson *B*-factor	26.94	55.14	36.34	52.89	45.35
R-merge	0.0639 (1.184)	0.05781 (0.7918)	0.09815 (0.8454)	0.08496 (1.018)	0.1086 (1.947)
CC1/2	1 (0.788)	0.999 (0.783)	0.999 (0.777)	0.999 (0.742)	0.999 (0.684)
R-work	0.1816 (0.2412)	0.2129 (0.3023)	0.2043 (0.2558)	0.2036 (0.2979)	0.1751 (0.3886)
R-free	0.2288 (0.2727)	0.2685 (0.3728)	0.2372 (0.3016)	0.2581 (0.4060)	0.2230 (0.4209)
*Number of non-hydrogen atoms*	1419	1205	1361	1204	1445
Macromolecules	1324	1205	1337	1204	1372
Ligands	4	0	0	0	20
Water	91	0	24	0	53
					
Protein residues	169	162	174	161	176
RMS (bonds)	0.006	0.009	0.005	0.009	0.007
RMS (angles)	0.87	1.20	0.66	1.25	1.05
Ramachandran favoured (%)	100	95	98	95	98
Ramachandran outliers (%)	0	1.3	0	1.9	0.59
*Average* B*-factor*	38.30	63.30	42.20	57.00	59.90
Macromolecules	37.70	63.30	42.30	57.00	59.70
Ligands	44.50	NA	NA	NA	85.70
Solvent	46.30	NA	36.10	NA	54.70

Statistics for the highest-resolution shell are shown within parentheses.

## Abbreviations

GFP: green fluorescent protein
MOM: mitochondrial outer membrane
SEC: size exclusion chromatography
SPR: surface plasmon resonance
